# Supporting close-to-community providers through a community health system approach: case examples from Ethiopia and Tanzania

**DOI:** 10.1186/s12960-015-0006-6

**Published:** 2015-03-28

**Authors:** Sarah Smith Lunsford, Kate Fatta, Kim Ethier Stover, Ram Shrestha

**Affiliations:** EnCompass LLC, 7200 Wisconsin Ave., Bethesda, MD 20814 USA; University Research Co, LLC, 7200 Wisconsin Ave., Bethesda, MD 20814 USA

**Keywords:** CTC providers, Community health systems, Improvement, Ethiopia, Tanzania

## Abstract

**Introduction:**

Close-to-community (CTC) providers, including community health workers or volunteers or health extension workers, can be effective in promoting access to and utilization of health services. Tasks are often shifted to these providers with limited resources and support from CTC programmes or communities. The Community Health System Strengthening (CHSS) model is part of an improvement approach which draws on existing formal and informal networks within a community, such as agricultural or women’s groups, to support CTC providers and address gaps in community-based health services. The model offers a framework for bringing representatives from existing community networks, CTC providers, and health facility staff together to form a community team charged with identifying challenges in service delivery, testing solutions, and monitoring changes. CTC providers draw upon fellow community team members to disseminate health messages and refer community members in need of services.

**Cases:**

Two cases are presented. In Ethiopia, the CHSS model was applied in 18 communities to increase HIV testing among pregnant women and antenatal care service utilization and improve sanitation. Prior to implementation, representatives from community groups were unaware of health extension workers or were uncomfortable making referrals. By participating on the community team, representatives became familiar with and comfortable referring people to health extension workers and spreading health messages. During implementation, more pregnant women registered for antenatal care and tested for HIV; health extension workers conducted more postnatal visits; and more households had functioning latrines and proper latrine use increased.

In Tanzania, the CHSS model was applied in five communities to improve HIV testing and retention into care. Community team members talked to their families and social networks about HIV testing and, when they identified someone who had dropped out of treatment, they referred those individuals to the home-based care volunteer. Increases in HIV testing and a reduction in patients lost to follow-up were observed.

**Discussion and conclusion:**

The CHSS model brings together existing networks within communities to support and lend legitimacy to CTC providers. This approach may result in sustainable community-based programmes, especially in HIV where the continuum of care extends beyond the facility and into the community.

## Introduction

There is a long history of community health workers (CHWs) and other close-to-community (CTC) providers in low-resource settings being utilized to meet development goals and relieve pressure on the professionally trained health workforce [1-4].

A wealth of literature has been written on the gains achieved through CTC provider programmes, especially those focused on child health—reducing malnutrition and neonatal and under-five mortality and providing community case management of childhood diseases. Success has also been noted in reducing maternal mortality and improving access to family planning services, disseminating insecticide-treated bednets for malaria control, and encouraging testing and delivering cost-effective, community-based treatment for tuberculosis [5]. For HIV prevention and care, CTC providers have been found to be effective in improving HIV-related knowledge [6], reducing risky sexual behaviours, increasing antiretroviral treatment (ART) uptake [7], providing home-based care, and improving access and quality of care [8].

CTC providers can offer much needed support to people living with HIV beyond what facility-based providers can deliver due to their proximity to their communities. They can play an instrumental role in linking communities with health care facilities, promoting ART adherence support, providing linkages to community-based services including income generation activities, and finding patients lost to follow-up.

However, CTC programmes face many challenges. The official status, extent of training, and compensation for CTC providers differ widely, depending on whether they are official government employees, volunteers, or are engaged through non-governmental organizations (NGOs). Payment can range from modest salaries [9,10] to performance-based incentives [11,12], community contributions [13], or incentives such as t-shirts or bicycles or non-material recognition [14,15]. Many, low- and middle-income governments have challenges paying salaried community workers and do not have the structure to absorb and pay or otherwise incentivize volunteers [1]. The productivity, motivation, and long-term sustainability of CTC providers may differ and be impacted by their level of compensation or incentives provided.

Because of the modest salary or incentives and the limited training provided, CTC providers are often viewed as a low-cost mechanism for responding to the shortage of professional health workers. Task-shifting responsibilities previously reserved for professional health workers to CTC providers have been found to efficiently and effectively improve access to health care commodities [16]. A systematic review of task shifting for HIV treatment and care in Africa found that task shifting can improve efficiency, access, quality of care, health outcomes, and relationships between facility-based staff and community-based workers [17]. However, as tasks continue to be shifted to these CTC providers, their workload grows [18], leading to a sense of feeling “overburdened” or “overworked” [12]. CTC providers may not have job descriptions at all, or even if they do have descriptions, these do not accurately reflect their expanding and evolving roles and responsibilities. Moreover, added tasks may not be supported by training or a supervisory structure [18], which in turn affects their retention and performance [8,19].

Selecting the correct individuals to function as CTC providers and ensuring they have the necessary training and support is essential to a successful CTC programme [20]. CHW performance, for example, requires supervision, support, and training to avoid undermining service quality [9,15,21]. Additionally, infrastructure, training, and support to minimize stock-outs of essential medicines among CTC providers are necessary [22]. Without these support systems, the sustainability of the services that CTC providers offer may be limited [8].

CTC providers also face challenges from their communities; they may not be accepted, recognized, or supported by the communities they serve if their selection is done without taking into account socio-cultural contexts. For example, in Afghanistan, the presence of a female CHW was found to improve use of modern family planning methods, antenatal care, and skilled birth attendance, while the presence of a male CHW did not have the same effect due to the socio-cultural norms that dictate how men and women interact [23]. Engaging communities in supporting CTC providers can have a positive effect. Receiving verbal feedback from communities and observing improvements in the health status of the communities that CTC providers serve can enhance their performance to a greater degree than supervisor feedback from the health facility [10].

These challenges faced by CTC programmes, CTC providers themselves, and the communities they serve can, in part, be addressed by applying the Community Health Systems Strengthening (CHSS) model developed by Ram Shrestha. This paper presents two cases which describe the application of the CHSS model to aid CTC providers in Ethiopia and Tanzania in fulfilling their responsibilities to improve the health and well-being of communities for HIV and other services. The work was funded by the U.S. President’s Emergency Plan for AIDS Relief (PEPFAR) through the United States Agency for International Development (USAID). These two cases represent applications of the model in two distinct health systems. In Ethiopia, the model was applied in a context of a strong government-supported CTC provider programme. In Tanzania, the model was applied in a context of a weaker volunteer CTC provider programme and was complementary to work being done at the facility level to expand improvement across the continuum of care.

### The CHSS model

Communities have existing systems for supporting one another through informal networks, such as coming together to assist families with weddings, during disasters or funerals, or through formal mechanisms such as women’s, agricultural, or savings and loans groups. The CHSS model presents a community-level framework that draws upon existing resources and activities to develop an infrastructure for continually and demonstrably improving the way that community groups can promote the health of community members and support the work of community health workers. The model utilizes the existing system within a community by bringing together representatives from networks and groups in an intentional way to support the CTC provider and achieve better health outcomes. This model provides a framework for leveraging and organizing existing community systems and networks, continuously improving health and social services offered at the community, and building the community relationship with the health facility. The CHSS model has been used to improve case identification, referrals and counter-referrals with the medical system, loss to follow-up, dissemination of health education messages, referrals to non-medical support services, and provision of general patient support [24].

Implementation of the CHSS model begins with project staff orienting leaders at the national, regional, and district levels to the approach and analysing existing links between the district, facility, and community levels. Facility and district staff in supervisory roles receive training in organizing community systems and leading quality improvement to become coaches and support community teams. Project, facility, and district coaches jointly visit each community to orient the community to the work and explore what groups and networks exist already.

**Figure 1:**
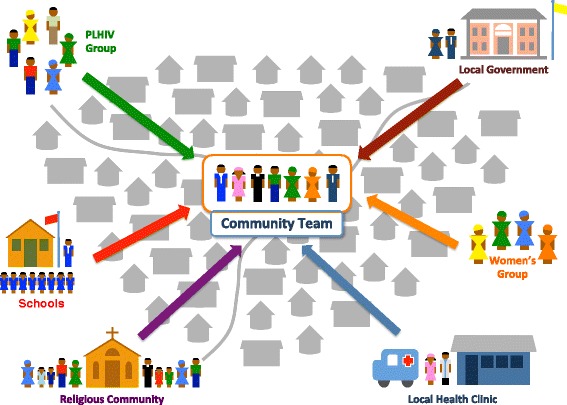
**The Community Health System Strengthening model.** The model brings representatives from various community groups to form a community team tasked with supporting the CTC provider and addressing gaps in health services delivered at the community level.

To begin the process of establishing a community team, coaches look for an existing health committee or similar structure which can serve as the anchor for these activities. Coaches negotiate with the existing health committee to add additional members from other community groups to ensure representation across the community for the purposes of improving a given health area (see Figure 1). While there have been variations in the composition of community teams across countries and communities, teams generally consist of CTC providers, health facility staff (as team members, coaches, and in many cases CTC provider supervisors), formal community leaders, community elders, religious leaders, and representatives of agricultural groups, savings and loans, and women’s organizations.

Coaches train and support community teams in their roles and responsibilities in identifying target groups, disseminating health messages, and applying principles of health care improvement, including working in teams, fostering people-centred care, examining processes for bridging care between the community and facility, identifying areas for improvement, conducting ongoing testing of potential solutions using rapid problem solving cycles, and monitoring interventions.

Following the initial training, community teams meet periodically; in the cases we describe below, they met either biweekly (Ethiopia) or monthly (Tanzania). These meetings are an essential component of problem solving cycles; members review data to determine gaps in performance, develop ideas to test, and determine when an idea was successful and should be implemented across the community or at a larger scale. Each team member is responsible for testing these ideas within their own network or group and households and bringing the results to the next meeting for discussion. Coaches support community teams through monthly visits. Additionally, in Ethiopia, teams from different communities came together every 4 to 6 months to share their progress and learning.

Two key tasks of the community team are to create or strengthen existing linkages between the community and the formal health system and to increase support for CTC providers across communities. Each community group representative is responsible for facilitating the transfer of health information and messaging to their network or group and bringing information, such as case identification, back to the CTC provider. Team members are not meant to provide these services solely or directly but rather as representatives who determine how their network or group can best play a role in supporting the CTC provider in one of the areas mentioned above. Together, the networks which they represent and have access to can reach the majority of the population more quickly and efficiently than a CTC provider alone.

The regular team meetings and coaching visits provide an opportunity for community representatives including CTC providers to share with and learn from facility and district representatives. This regular interaction between CTC providers and facility and district staff, who often function in a supervisory capacity, can provide space to strengthen their professional relationships. It also creates an environment for more supportive supervision, open communication, and enhanced appreciation of CTC providers in the health system. Community team members who represent other community groups also get to know the CTC providers and facility and district staff through these regular meetings, strengthening the community-CTC provider-facility linkages. These improved relationships borne out of community team membership and cooperation can make representatives of community groups more aware of CTC providers and the services they provide and make the community team members more comfortable in referring community members to the CTC providers for care.

While the community health system itself does not directly address larger systemic issues such as supply chain or infrastructure, data and experiences generated by the community teams can contribute evidence highlighting gaps in the larger system. The coaching support structure creates the opportunity for regular interaction with coaches and allows these representatives from higher levels of the health system to see the reality on the ground and advocate for making changes to address gaps such as supplies, access, or human resources.

Health conditions such as HIV and services such as antenatal care (ANC) and basic sanitation require a care and support model that extends beyond the health facility and into the community. In the context of HIV, disengagement in care and treatment can occur along the entire continuum of care among adult patients [25] and in the prevention of mother-to-child transmission of HIV [26]. Social and institutional dynamics, including CTC providers, can help promote regular counselling and testing and retention into care, mitigating losses [25].

### Case studies

#### Ethiopia

In Ethiopia, research found that health extension workers (HEWs) spent their time moving from house-to-house, leaving the health posts closed and thus underutilized [27]. The Federal Ministry of Health requested the USAID Health Care Improvement Project (HCI) to provide support to bolster linkages between communities and the health system, improve HEW effectiveness, and improve the capacity of community groups to take ownership of health programmes, focusing on HIV and hygiene. From November 2011 to September 2012, USAID HCI supported community-based teams in 18 *kebeles* (communities) served by three health centres in Illu and Tole *woredas* (districts) in Ethiopia’s Oromia Region.

HEWs are paid government CTC providers located at community-level health posts. They are responsible for a basic package of 16 services, including immunization, treatment of common illnesses, basic maternal and newborn care, sanitation, and health education. Each HEW is responsible for 2,500 people. They are supervised administratively by the *woreda* health office and clinically by the health centre. HEWs face barriers to effective performance, including lack of supervision, supplies of drugs and equipment, clear referral systems, transportation, and communication systems [28]. Before the introduction of the CHSS model, HEWs travelled from house-to-house to identify pregnant women and provide basic ANC services, but they were unable to reach every household. As a result, many pregnant women were missed and did not receive services.

USAID HCI staff began by orienting regional, district, and health centre personnel to the CHSS model and improvement methods and selected 20 supervisors from each level to serve as coaches. Following a 3-day training on improvement and data management, the coaches carried out situational analyses to identify and map community groups, their networks, other sector groups, village leaders, schools, volunteers, and other health agents. Coaches, along with an advisor from USAID HCI, held meetings with community representatives in each health post catchment area and discussed the purpose of the activity and role of the community team. The community team was formed from an existing village committee, *kebele* managers, HEWs, health centre staff (as coaches), religious leaders, and government development agents. If any group identified during the situational analysis did not have representation in this community team, members were added.

After the situational analysis was completed by the community teams, they determined to focus on improving ANC visits, HIV testing among pregnant women, postpartum visits, and sanitation. The teams emphasized identifying and referring those in need of services to HEWs, ensuring households constructed and used pit latrines, and mobilizing the community to remove stagnant water. Using the CHSS model, each team developed a clear process for members of each group to spread messages about ANC services and communicate information about new pregnancies to the HEW through the team members. The HEW was able to take information from the groups and compare that to data on women who had gone to the health facility for ANC services. The team used the reach of their groups to encourage building latrines. Informal structures, like *iddir* (voluntary association), provided forums for HEWs to deliver health messages and offered support to households around reducing health risks and seeking HEW services.

With the introduction of the CHSS model, pregnancy identification and women receiving their first ANC visit increased immediately. For example, nine communities in the Illu *woreda* reported that they had identified 103 women in the first month following the start of the intervention, 72% of whom registered for ANC at the health post. Over the course of 10 months, they identified 259 pregnant women, 86% of whom had registered for ANC. There was an initial spike of identifications in the first month as the community team identified all currently pregnant women, followed by a steady stream of information about newly pregnant women. Other results include the following: an increase in the proportion of households with latrines from 30% to 60% in the Golole *kebele*, an increase in proper latrine use from 36% to 76% of households in the Tulu Mangura *kebele*, an increase in the proportion of pregnant women referred by an HEW and tested for HIV from 55% to 86% in six kebeles in Tole district, and an increase in post-natal women visited by a HEW in nine *kebeles* in the Illu district from 74% to 91% of women identified by the community.

In September 2012 at the end of the intervention, qualitative interview data were collected in 3 of the 18 participating *kebeles* to explore team members’ experiences with the model. The results reflected that relationships across the community had been strengthened and that HEWs felt better supported. Support to HEWs was perceived as improving during the intervention as members of the community team and other community group leaders took greater responsibility for providing HEWs with feedback and linking the community with their services. Community team members felt more accountable to their fellow community members; one expressed that prior to joining the community team he did not know how to respond to community members’ requests for advice on health issues. Being part of the team made him confident in referring individuals to HEWs. By strengthening these links, HEWs felt more connected and, in their view, more effective: As stated by one HEW, “Previously the community was not convinced that I could indeed help them with their health problems. Now they are convinced that not only me, but…team members could also contribute to their own health”. Clients shared that they, too, were more comfortable with HEW services. According to one client: “The HEW is like our friend. I do not find it difficult to share every problem I have with her; she either helps me or takes me to the health center”.

HEWs also felt their reach had increased: One HEW stated, “There is not member of a household who cannot be reached now. Each team knows who is pregnant, who is lactating, who has a latrine, who sleeps under an [insecticide treated bednet]”. Zone Health Department staff agreed. One stated: “This is a cost-effective and innovative initiative. Performance…as concerns maternal health is 60% but in [intervention] *kebeles*, our performance shows nearly 100%”.

HEWs indicated that prior to the intervention there was no mechanism for identifying obstacles in service delivery like lack of medicines and supplies. Community team meetings offered a venue to raise challenges and identify solutions with representatives from the facility and district, including those challenges that required a system-wide solution. However, not all HEW needs were able to be met. Limited support to HEWs, specifically lack of in-service training, hindered developing practical skills. According to one HEW supervisor, “training is organized based on decisions at a higher level”, and neither HEWs nor their supervisors were involved.

#### Tanzania

From January 2014 to August 2014, the USAID Applying Science to Strengthen and Improve Systems (ASSIST) Project worked with the Council Health Management Teams to apply the CHSS model in five communities in Muheza District of Tanga Region, Tanzania. The aim was to increase uptake of HIV testing and reduce loss to follow-up of patients on ART through improved linkages between health facilities and the communities. Previous work under the USAID ASSIST Project had focused on improvement at the facility level; however, more needed to be done at the community level to retain patients in care. USAID ASSIST chose communities that were within the catchment area of facilities that received technical support.

Prior to applying the model, government-endorsed home-based care (HBC) volunteers chosen from within the communities were the primary link between the facility and the community. HBC volunteers were responsible for covering 20–25 households, which made it difficult to reach all households on a regular basis with the full package of health information and basic services they were responsible for delivering. A district-level HBC coordinator supported the HBC volunteers but had limited interactions with them, meeting only when HBC volunteers were at the local health facility as opposed to providing supportive supervision in the community. Health facility staff interacted with HBC volunteers and with only those patients who came to the clinic. Facility staff attempts to reach patients who were lost to follow-up were limited to telephone calls to patients with known mobile numbers and resulted in limited to no success.

USAID ASSIST and district-level staff identified active groups and committees in each community. Members were invited to trainings to discuss HIV care, including adherence to treatment and loss to follow-up, educating others, and advantages and disadvantages of HIV testing. During the trainings, the CHSS model was introduced, including improvement principles and rapid problem solving, and a team was formed in each community. In most cases, the teams consisted of the existing village health committee plus additional representatives from various community groups and the HBC volunteer. This team discussed the current status of HIV in their community, including reviewing facility data on HIV testing and loss to follow-up. The team met monthly to review data, discuss whether they were accomplishing their aim, and decide on immediate actions they could take to improve the processes.

USAID ASSIST worked with the district to identify appropriate coaches to support the community teams, including the district HBC coordinator, health centre and dispensary HBC focal people, a district social welfare officer, an agricultural extension officer, and a community development officer. These coaches were trained in how to organize the community system and facilitate quality improvement. In each village, one HBC volunteer was also trained to lead the community team. USAID ASSIST hired a Community Systems Strengthening Coordinator who lived in the area to provide intensive initial support to communities. A few coaches visited the communities each month to support and participate in the community team meeting.

The team designed a new process through which information about health would be communicated to community members. Each team member brought health messages from the HBC volunteer to the regular meeting of the committee or group they represented. Members of those groups were encouraged to talk to their family members, both HIV positive and negative, about the messages, such as the importance of HIV testing and adherence and continuation of ART treatment. This process was viewed as providing more rapid dissemination of information than the HBC volunteer could accomplish alone. Members of the People Living with HIV (PLHIV) group reported receiving health messages from the community team.

To improve retention in treatment, the HBC volunteer reviewed facility lost-to-follow-up data with the community team; to maintain confidentiality, only the number of patients who were lost was discussed, not individual identities. The team then shared messages on the importance of remaining on treatment with the other community groups. When patients who had dropped out of treatment were identified by community team members through their family or close social networks, they were connected with the HBC volunteer who further educated and encouraged them to return to treatment and connected them back with the facility. The community team also connected patients who had left treatment to the PLHIV group in the community which offered a support network and, in some cases, opportunities for income-generating activities for the patients.

Based on self-reported facility data covering the five communities, 106 people were tested for HIV in January 2014 (42 men and 64 women). By February 2014, that number increased to 319, 50 of whom were returning for a follow-up test. While community-based testing was an activity facilities carried out periodically, for it to be successful, there needed to be sufficient awareness and demand from the community. The community team was able to disseminate messaging and create the demand, resulting in high-testing rates in February 2014. Not only did the number of people tested for HIV increase, but the number of male partners who came for testing HIV also increased. By June 2014, the number of individuals being tested normalized following the initial spike, with 133 people tested that month.

In addition to more individuals being tested, other aspects of testing services were improved. One community group member, a representative from the PLHIV group, noted that more people being tested were returning to the facility to receive their results than before the activation of the community teams. Confidentiality around test results was also maintained, further motivating community members to get tested, according to a community team member. After the initial spike in testing, community teams continued to share messages on the importance of HIV testing and retesting every 3 months.

At the beginning of March 2014, 31 patients were lost to follow-up, and from March to September 2014, an additional 13 were lost. By the end of September 2014, of these 44, 23 patients were back on treatment, 5 had relocated, 11 were identified as having died, and 5 were still lost. A community team member observed that “if we only use the HBC volunteer, we won’t get the lost-to-follow-up. But, if we use this model with the HBC, we will get them back”.

During interviews, community teams reported that seeing people going to test for HIV made them feel that they were having a positive impact on the health of their families and the wider community. HBC volunteers expressed relief that they were no longer working in isolation; they had a network through which they could spread messages and a team of people with whom they could problem-solve. One HBC volunteer commented that “information doesn’t stop now, it flows. The community used to be far from the facility, now it is close”.

HBC volunteers felt that their communities had an improved understanding of HIV and overall health. They noted that before this work, only pregnant women and people who were feeling ill would be tested for HIV; following the intervention, men and women were being tested regardless of their current health status. Facility providers indicated that they saw new multi-sectoral involvement and increased service utilization. They offered that they could not have achieved this on their own. The district HBC coordinator commented that she began to see motivation in the communities that was not there previously. She also began attending community team meetings in addition to conducting facility visits. She said that she had previously heard about community mobilization and engagement but she never saw it until implementation of the model which led, in her view, to true community engagement. Follow-up conversations with participating communities in February 2015, 4 months after the conclusion of USAID ASSIST technical support, revealed that community teams continued to meet regularly to identify and address additional areas for improvement.

## Discussion and conclusion

We have presented two case studies of the application of the CHSS model in Ethiopia and Tanzania as a mechanism for strengthening the effectiveness of CTC providers in promoting retention in HIV care, access to ANC, and improvements to sanitation services. These cases illustrate how working with existing community groups and networks can create a system of intentional support for CTC providers. Representatives from various community groups, together with health facility staff representatives, district health staff, and CTC providers, collaborated to improve the health of community members.

The case studies discussed highlight how the application of the CHSS model can contribute to overcoming existing barriers to the HIV continuum of care. In both countries, community team members each had their own social networks and could educate them on the importance of HIV testing and retention into care. The messaging from a familiar and credible source may have inspired more community members to seek services, contributing to making health promotion more people-centred. Community team members also lent credibility to the CTC providers, encouraging community members to follow advice and referrals from CTC providers, thereby reducing loss to follow-up at all points in the continuum of care. However, this model can be equally beneficial in working towards other health or social services goals.

Application of the CHSS model was shown to be effective in diffusing workload among a variety of actors and increasing reach. Specifically, it was shown that existing groups and communication networks in communities can contribute to health promotion, education, awareness-raising, mobilization, case identification, referrals, and follow-up, allowing the CTC focus on other services. This created the potential to increase CTC provider productivity and retention and promote efficiency and effectiveness of the overall health system, which could be the subject of additional research. In addition, the new way of working through existing groups and networks is more likely to be sustained as it does not rely on one trained individual to carry forward the knowledge of how to work through these communication and referral networks.

By engaging with and providing support to CTC providers, community improvement team members helped to increase community members’ confidence in CTC providers. A valuable trust relationship [29] was created between team members and the CTC provider so the team members were not reluctant to publicly endorse and refer people to the CTC provider. The status of the CTC provider was raised, making the community more aware of the benefits and services they can provide and more willing to access those services. Through the community team, the community became more engaged in working with the CTC provider to identify and remove barriers to care.

The community team provided a platform for CTC providers and facility staff to engage regularly, build a relationship, and thus enhancing facility staff recognition of the CTC providers as members of the formal health system. By coaching the community team, facility and district staff became more in touch with the realities and constraints experienced by CTC providers as evidenced by the Muheza District HBC coordinator in Tanzania who began engaging with the community more regularly as a result of participating in the model application. The CHSS model also has the potential to strengthen government-mandated community-level structures, such as the village health committee in Tanzania.

This paper illustrated how the CHSS model offers an alternative approach to improving the effectiveness of CTC providers; however, there are some considerations to be taken into account when applying this model. First, successful application requires an understanding of specific community context. This knowledge is necessary to identify existing community groups who should be represented on the community team. Awareness of the community context is also essential to facilitate implementing the approach in such a way as to be responsive to the informal structures, community needs, and existing relationship with the formal health system and, importantly, CTC providers’ needs. It must also be noted that the formal and informal networks the model draws upon may be stronger in rural communities than in urban settings where they may be fractured due to internal migration and urbanization [30]. Related is the importance of understanding how the CTC provider programme functions in the context of the larger health system. In Mozambique, for example, research indicates that many government-supported CTC providers had been inactive due to programming challenges, policies, and resource limitations [31]. Thus, one of the early activities of the community teams was to activate and build the recognition of the CTC providers within the community. Knowing both the community context and how CTC providers fit within the health system as a whole is an essential first step in applying the CHSS model.

A second consideration is the need for sufficient resources to form community teams and build their capacity to collect and use data, identify problems, and test and implement potential solutions. These resources include coaching on how to identify gaps and resolve them and financial and logistical support for coaching visits and training participation. Experience in applying this model in the two cases described here, Ethiopia and Tanzania, as well as applications in other countries has taught us that it is best to ensure these resources are made available in such a manner as to build a foundation of collaboration. No additional funds were supplied by the USAID HCI or USAID ASSIST Projects to pay community team members which may encourage sustained work by the community team.

Third, it is important to consider which issues the community team will address first. We recommend starting with simple or more easily addressed issues while community teams are learning this new way of working with each other and using improvement strategies. This allows the teams to build their skills and confidence before moving on to more complex challenges.

Finally, it must be recognized that the application of the CHSS model cannot meet every challenge faced by CTC providers, such as insufficient training or gaps in the supply chain. We recommend the CHSS model be part of a larger health systems strengthening approach to build the functionality of the system at all levels. This should include formal recognition of the value and contribution of CTC providers.

The case studies presented here have some limitations that should be noted. Indicator data were self-reported, and there was no verification on the validity or accuracy of the data. Qualitative data for these case studies were collected by a member of the project team, which may have introduced social desirability bias. While some information was gathered from the communities in Tanzania following the conclusion of USAID ASSIST support, we were not able to follow these communities and the community teams to observe sustained work or results, though this would be an important area for future research.

The cases presented here illustrate a promising approach to engaging existing community networks and mechanisms, both formal and informal, to support CTC providers in fulfilling their mandates and improving the health outcomes of communities.

## Abbreviations

ANC: Antenatal care
ART: Antiretroviral therapy
ASSIST: USAID Applying Science to Strengthen and Improve Systems Project
CHSS model: Community Health Systems Strengthening model
CTC: Close-to-community
HBC: Home-based care
HEW: Health extension worker
PEPFAR: U.S. President’s Emergency Plan for AIDS Relief
PLHIV: People Living with HIV
USAID: United States Agency for International Development
